# Strychnine in Sciatica

**Published:** 1858-11

**Authors:** O. C. Gibbs

**Affiliations:** Frewsburg, Chautauque Co., N. Y.


### ART. III.—*Strychnine in Sciatica.* By O. C. Gibbs, M. D., Frewsburg, Chautauque Co., N. Y.

In the September number of the American Medical Monthly, for 1857, I reported a case of sciatica promptly cured with strychnine, after a failure of a great variety of other remedies usually resorted to in such case. The patient once before had suffered from a similar attack, which yielded only after a year and a-half of thorough treatment at the hands of a skillful and intelligent physician. In the attack which came under my observation, colchicum, ammoniated tincture of guiacum, quinine and morphia, oil of turpentine, tinct. of cimicifuga, iodide of potassium, Dover's powders, blisters over the great trochanter, the endermic application of morphia, cups over the same part, and calomel to touch slightly the gums, had all been put to the test and failed to afford relief. Four weeks had been spent in the trial of the remedies above mentioned, with no other effect than to impair the general health. Strychnine was now brought to bear upon the case, in doses of one-sixteenth of a grain, with immediate and decided effect; a cure was perfected in two weeks, which, up to the present time, after a lapse of a year and a-half, remains permanent.

Since the case above referred to was reported, I have treated one other case with strychnine with similar, though with not quite as decided results. The following is a brief synopsis of the case:

*April* 10, 1858. I was called to see Mr. E., aged about 60 years. He laboring under extreme pain in one hip. The pain was particularly severe between the great trochanter and the ischium, extending thence downward to the knee. The patient had been suffering about two months; having a holy horror of "allopathic" physicians, who some times administer poisons, he had taken only patent medicines. The patient was somewhat anæmic, and I consequently ordered two grains of quinine and five of Dover's powders, three times a-day; also drachm doses of ammoniated tincture of guiacum three times a-day. This treatment was continued for three weeks without improvement. I now ordered

℞ Tinc. colchicum, ℥j.

Tinc. cimicifuga, ℥ij. M.

Dose a teaspoonful three times a-day instead of the ammoniated tincture of guiacum; the quinia and Dover's powders to be continued.

This treatment was continued for three weeks longer with no apparent benefit. I now ordered two grains of strychnine, in crystals, to be put with two ounces of water, slightly acidulated with aromatic sulphuric acid, of this half a teaspoonful was to be taken three times a-day, the quinia and Dover's powders to be continued.

From this date improvement commenced, and steadily continued, until the patient was discharged two weeks subsequently, after taking about six grains of strychnine.

About two weeks after he was discharged I saw the patient again; he was in comfortable health, but complained of slight stiffness in the hip, and occasional slight pain. He said he thought if I had given him about two grains more of strychnine, his recovery would have been perfect.

Up to the present time, three months since discharged, he has suffered no return of the disease.

